# Potential of Human Norovirus Surrogates and *Salmonella enterica* Contamination of Pre-harvest Basil (*Ocimum basilicum*) via Leaf Surface and Plant Substrate

**DOI:** 10.3389/fmicb.2018.01728

**Published:** 2018-07-30

**Authors:** Dan Li, Mieke Uyttendaele

**Affiliations:** ^1^Food Microbiology and Food Preservation Research Unit, Department of Food Technology, Safety and Health, Faculty of Bioscience Engineering, Ghent University, Ghent, Belgium; ^2^Food Science and Technology Programme, Department of Chemistry, Faculty of Science, National University of Singapore, Singapore, Singapore

**Keywords:** norovirus, *Salmonella*, basil, fresh produce, internalization

## Abstract

Fresh produce has been identified as an important vehicle for foodborne pathogen transmission and fresh culinary herbs have occasionally been associated with human pathogens and illness. In this study, the fate of human NoV surrogates [murine norovirus 1 (MNV-1) and Tulane virus (TV)] and three strains of *Salmonella enterica* on pre-harvest basil (*Ocimum basilicum*) was investigated. The persistence after contamination via either leaf surface or plant substrate was tested respectively. After 3 days, both MNV-1 and TV on pre-harvest leaves were at non-detectable levels (>5.5-log reduction for MNV-1 and >3.3-log reduction for TV). The three *Salmonella* strains showed consistent reductions of 3- to 4-log. At day 6 and 9, all the tested samples showed low levels of infectivity which were close or below the detection limits (1.7-log PFU/sample leaf for MNV-1 and TV, 0.7-log CFU/sample leaf for *Salmonella*) except for *S.* Thompson FMFP 899, one out of three samples showed to maintain present at exceptional high levels (day 6: 5.5-log CFU/sample leaf; day 9: 6.7-log CFU/sample leaf). Possibilities of microbial internalization into the edible parts of basil via the roots was demonstrated with both MNV-1 and *S. enterica* Thompson FMFP 899. The infectivity of internalized MNV-1 and *S. enterica* both decreased to non-detectable levels within 9 days after inoculation. Moreover, it should be noticed that very high microbial inoculation was used in the experimental set-up (8.46-log PFU/ml of MNV-1, 8.60-log CFU/ml of *S. enterica*), which is abnormal in the real-life expected contamination scenario. Within the tested scenarios in this study, *S. enterica* contaminated on the adaxial leaf surface of basil plants while in growth, and remained/reached a high population of over 6-log CFU/sample leaf after 9 days in one out of three samples, thus showed the highest potential for causing foodborne infection.

## Introduction

Fresh produce has been identified as an important vehicle for the transmission of foodborne pathogens including *Salmonella*, Shiga toxin-producing *E. coli* (STEC), *Listeria monocytogenes*, human noroviruses (NoVs), Hepatitis A virus, etc. (Li et al., 2015; Alegbeleye et al., 2018). Contamination can occur during pre-harvest, harvest and post-harvest stages. In general, the pre-harvest contamination comes mainly from fertilizer or irrigation water. It was observed that the pathogens not only survive on the surface of fresh produce, but can also be internalized into the plant tissues via the roots (Deering et al., 2012; Erickson, 2012; Hirneisen et al., 2012; Alegbeleye et al., 2018).

Fresh culinary herbs have occasionally been associated with human pathogens and illness (FDA, 2013). They have drawn special attention on food safety since once fresh culinary herbs are contaminated, completely removing or killing pathogens is unlikely (FDA, 2013). On the other hand, however, anti-microbial activities of herb and spice compounds have been reported against foodborne pathogens such as *Salmonella* Typhimurium, *Escherichia coli* O157:H7, *Listeria monocytogenes*, *Bacillus cereus*, and *Staphylococcus aureus* (Tajkarimi et al., 2010). Therefore, it is of interest to investigate the fate of foodborne pathogens once they contaminate culinary herbal plants, both via leaves and roots, in order to provide data for comprehensive risk assessments.

Basil (*Ocimum basilicum*) is a worldwide popular and commonly used culinary herb. In this study, the fate of human NoV surrogates [murine norovirus 1 (MNV-1) and Tulane virus (TV)] and three strains of *Salmonella enterica* on pre-harvest basil leaf surfaces were investigated. Tests on fabric fake basil leaves were done as a control. The internalization of the MNV-1 and *Salmonella* into edible parts of basil from the roots was also evaluated.

## Materials and Methods

### Virus Preparation

Cells of the murine macrophage cell line RAW 264.7 (ATCC TIB-71) and LLC-MK2 (ATCC CCL-7) were maintained in complete Dulbecco modified Eagle medium (DMEM) and grown at 37°C under a 5% CO_2_ atmosphere. Complete DMEM consisted of DMEM (Lonza, Walkersville, MD) containing 10% low-endotoxin fetal bovine serum (HyClone, Logan, UT, United States), 100 U/ml penicillin, 100 μg/ml streptomycin (Lonza), 10 mM HEPES (Lonza), and 2 mM L-glutamine (Lonza).

MNV-1 was prepared by infecting of RAW264.7 cells with MNV-1.CW1 (kindly provided by H. W. Virgin, Washington University School of Medicine, St. Louis, MO, United States), passage 7, at a multiplicity of infection of 0.05 (MNV-1:cells) for 2 days. TV was prepared by infecting of LLC-MK2 cells with TV (kindly provided by X. Jiang, Cincinnati Children’s Hospital Medical Center, Cincinnati, OH, United States), passage 3, at a multiplicity of infection of 2.5 (TV:cells) for 2 days. After three freeze-thaw cycles, low-speed centrifugation was used to remove cellular debris from the virus lysate. The lysate was stored in aliquots at −75°C.

### Virus Titration by Plaque Assay

The titers of MNV-1 and TV were determined by plaque assay. Briefly, cells (RAW 264.7 cells for MNV-1 and LLC-MK2 cells for TV) were seeded into six-well plates. On the following day, when the cells were ∼80% confluent, 10-fold dilutions of the samples of unknown virus titer were prepared in complete DMEM, and 1 ml per dilution of the sample was plated onto two wells (0.5 ml per well). The plates were incubated for 1 h at room temperature and manually rocked every 15 min before aspirating the inoculum and overlaying the cells with 1.5% Sea-Plaque agarose (Cambrex, Rockland, ME, United States) in minimum essential Eagle medium (MEME; Lonza) supplemented with 10% low-endotoxin fetal bovine serum, 1% HEPES, 1% penicillin-streptomycin, and 2% glutamine (complete MEME) per well. The plates were incubated at 37°C and 5% CO_2_ for 2 days for MNV-1 and 3 days for TV. To visualize the plaques formed by MNV-1, RAW 264.7 cells were stained with 1.5% SeaKem agarose in complete MEME containing 1% neutral red (Sigma- Aldrich, St. Louis, MO, United States) per well for 6 h. To visualize the plaques formed by TV, LLC-MK2 cells were fixed with 3.6% formaldehyde [Sigma-Aldrich; diluted in phosphate-buffered saline (PBS, pH 7.5, Lonza)] for 30 min. The agarose-medium overlays were removed and the cells were stained with 0.1% (w/v) crystal violet (Sigma-Aldrich; diluted in 10% ethanol).

### Bacterial Strains

The *Salmonella enterica* serovar Typhimurium strain SL 1344 was a reference strain and the *Salmonella enterica* serovar Thompson strain RM1987 was isolated from cilantro. Both strains were obtained from Dr. Maria Brandl (U.S. Department of Agriculture, Agricultural Research Service, Albany, CA, United States). The *Salmonella enterica* serovar Thompson strain FMFP 899 was isolated from basil (Delbeke et al., 2015a).

### Plant Cultivation

Seeds of sweet large leaved basil (*Ocimum basilicum*) were purchased from an online seeds supplier “Seeds4garden” (Sluis Garden, the Netherlands) and germinated on 1% distilled water agar in petri dishes at 22°C in darkness. After 5 days germination, the seedlings were transplanted onto tap water soaked germinating discs (three seedlings per disc; JIFFY, the Netherlands) in a four-pot hydroponic system (four discs per 11 L-pot filled with clay pebbles; Wilma, United Kingdom) in an indoor grow box (Mammoth Lite 90, Netherlands). A 250W lamp (SONLIGHT AGRO) was used in the grow box to supply light with a photoperiod of 18 h and a dark period of 6 h. Nutrition solutions (30 ml of solution A and 30 ml of solution B; Bcuzz Hydro, Alami, Netherlands) were mixed with 30 L of tap water in the hydroponic system and supplied to irrigate the plants for two times of half hour each day by a water pump. After 2 weeks, an extra 20 ml of solution A and 20 ml of solution B mixed with 10 L of tap water were added in the system. The temperature (24 ± 2°C) and relative humidity (43 ± 5%) were monitored during the full growth period by an EL-USB-2-LCD+ logger (Lascar Electronics Ltd., United Kingdom).

### Adaxial Leaf Surface Inoculation and Detection

Virus or bacteria suspensions were inoculated by pipetting onto the adaxial leaf surfaces of the 4 week old plants in the grow box. For each leaf, 50 μl of virus or bacteria suspension was distributed evenly in a 1 cm^2^ square area labeled by a marker pen. In parallel, fake basil plants made from fabric were inoculated in the same way as a control.

Three, six or nine days after inoculation, the marked leaves were removed from the plants, the 1 cm^2^ inoculated squares were cut and put in extraction bags with filters (Bioreba, Switzerland) separately (one sample per bag). Five ml of PBS (pH 7.5, Lonza) was added in each bag. A homogenizer hand model (Bioreba, Switzerland) was used to grind the tissues, and the filtrations were collected. The virus samples were stored at −75°C before the virus titration by plaque assay. The bacterial samples were plated on selective media Xylose-Lysine-Desoxycholate Agar (XLD, Oxoid).

### Plant Substrate Inoculation and Detection

Each germinating disc with 6 weeks old basil plants (therefore the lower part of the roots, three plants per disc) was soaked in 10 ml of MNV-1 lysate (8.46 log-PFU/ml, eight discs in total) or 24 h culture of *Salmonella enterica* serovar Thompson strain FMFP 899 (8.60 log-CFU/ml, eight discs in total) in Tryptone Soya *Broth (TSB, Oxoid)* in a 100 ml sterile beaker for 1 h in the grow box. Afterward each day, 10 ml of nutrient solution as described above [30 ml of solution A and 30 ml of solution B (Bcuzz Hydro, Netherlands) mixed with 30 L of tap water] was added to each beaker to maintain the growth of basil plants.

At day 0 (1 h after inoculation), day 1, day 3, day 6, and day 9, three samples of 2 g basil leaves and shoots (edible parts) were collected from the plants grown on the eight discs (24 plants for MNV-1 test and 24 plants for *Salmonella* test in total) randomly. For MNV-1, 2 ml of PBS was added to the 2 g plant tissue in a bag with filter (Bioreba) and grinded by a homogenizer hand model (Bioreba) The filtrates were stored at −75°C before the virus titration by plaque assay. For *Salmonella* FMFP 899, 2 ml of Buffered Peptone Water (BPW, *Oxoid)* was added to the 2 g plant tissue in a bag with filter (Bioreba) and grinded by a homogenizer hand model (Bioreba). Pre-enrichment quantification: one ml of the filtrate was plated directly on XLD agar (two 90 mm petri dishes for each sample) and incubated at 37°C for 24 h. Post-enrichment detection: 10 ml of BPW was added to the rest of plant tissues in the bag and incubated at 37°C for 24 h. The enriched culture was streaked on XLD agar. After incubation at 37°C for 24 h, typical black colonies were confirmed by the BBL CRYSTAL^TM^ Enteric/Non-fermenter (E/NF) Identification (ID) System (Becton Dickinson, Franklin Lakes, NJ, United States).

## Results

**FIGURE 1:**
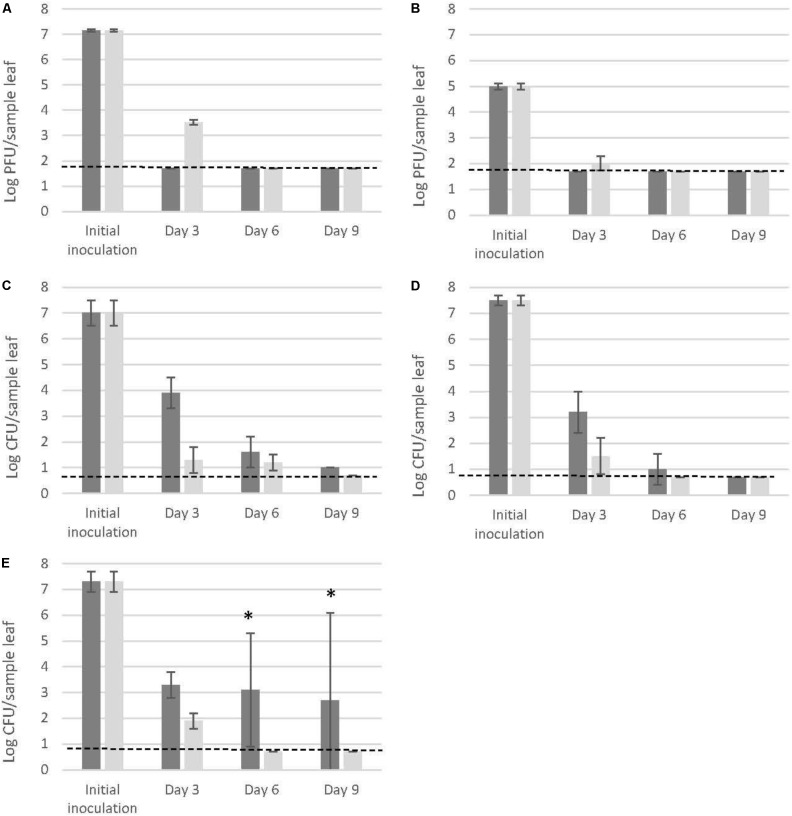
Detection of MNV-1 **(A)**, TV **(B)**, and three *Salmonella enterica* strains [**(C)**. Typhimurium SL 1344, **(D)** Thompson RM1987, **(E)** Thompson FMFP 899] on pre-harvest basil leaves (dark gray bars) and on fabric leaves (light gray bars). Each column represents the average of triplicates, and each error bar indicates the data range. Horizontal broken lines indicate the detection limits (1.7-log PFU/sample for MNV-1 and TV, 0.7-log CFU/sample for *Salmonella*). ^∗^One out of three samples at high levels (day 6: 5.5-log CFU/sample; day 9: 6.7-log CFU/sample), two out of three samples at low levels near the detection limit.

Three days post inoculation on the adaxial leaf surfaces of the 4 week old pre-harvest basil plants, both MNV-1 and TV were at non-detectable levels, corresponding to >5.5-log reductions for MNV-1 (**Figure 1A**) and > 3.3-log reductions for TV (**Figure 1B**) of the initial inoculum levels. The three *Salmonella* strains showed consistent reductions of 3 to 4-log (**Figures 1C–E**). At day 6 and 9, all the tested samples (MNV-1, TV and *Salmonella*) showed low levels of infectivity/enumeration which were close or below the detection limits (1.7-log PFU/sample leaf for MNV-1 and TV, 0.7-log CFU/sample leaf for *Salmonella*). The only exception was noted for *S.* Thompson FMFP 899: one out of three samples showed high levels of surviving cells (at day 6: 5.5-log CFU/sample leaf; at day 9: 6.7-log CFU/sample leaf, **Figure 1E**).

Higher viral reductions were observed on pre-harvest basil leaves than on fabric leaves after 3 days (MNV-1: > 5.5-log reduction on pre-harvest basil leaves vs. 3.6 ± 0.1-log reduction on fabric leaves, **Figure 1A**; TV: > 3.3-log reduction on pre-harvest basil leaves and 3.0 ± 0.3-log reduction on fabric leaves, **Figure 1B**). On the contrary, the three *Salmonella* strains showed higher reductions on the inert surface than on pre-harvest basil leaves at day 3, 6, and 9 without exception (**Figures 1C–E**).

Possibilities of microbial internalization into edible parts of basil via the roots was demonstrated with both MNV-1 and *S. enterica* Thompson FMFP 899. Edible tissues (mainly leaves) of growing basil plants were sampled to measure the presence of MNV-1 and *S. enterica* Thompson FMFP 899 at day 0 (1 h), day 1, day 3, day 6, and day 9 after inoculation. For MNV-1, infectious viruses were detected at day 1 (540, 580, 400 PFU/g tissue) and day 3 (280, 80, < 4 PFU/g tissue) by direct virus titration of the plant tissues filtrations with the use of plaque assay (**Table 1**). For *S. enterica* Thompson FMFP 899, no typical colony was observed by direct enumeration of the plant tissues filtrations on XLD agar ( < 2 CFU/g tissue) although after 24 h enrichment, one out of three samples at day 3 and day 6 showed typical black colonies on XLD agar (**Table 1**). These colonies were randomly picked to be confirmed by the BBL CRYSTAL^TM^ Enteric/Nonfermenter (E/NF) Identification (ID) System (Becton Dickinson, Franklin Lakes, NJ, United States) and showed confidence factors of 0.997 as *Salmonella* species.

**Table 1:** Detection of MNV-1 and *Salmonella* FMFP 899 from edible tissues of basil plants in growth inoculated via plant growing medium.

	MNV-1	*Salmonella* FMFP 899	
	Plaque assay	Pre-enrichment quantification	Post-enrichment detection
Day 0	<4 PFU/g tissue	<2 CFU/g tissue	0/3
Day 1	540, 580, 400 PFU/g tissue	<2 CFU/g tissue	0/3
Day 3	280, 80, < 4 PFU/g tissue	<2 CFU/g tissue	1/3, confirmed
Day 6	<4 PFU/g tissue	<2 CFU/g tissue	1/3, confirmed
Day 9	<4 PFU/g tissue	<2 CFU/g tissue	0/3

## Discussion

Both NoVs and *Salmonella* are leading foodborne pathogens worldwide which can be transmitted by fresh produce. For human NoVs, although there have been recent breakthroughs reported in tissue culture models (Jones et al., 2014; Ettayebi et al., 2016), none of them are feasible for routine food and environmental testing due to the presence of residual food matrix components as well as the cost- and labor implications. Therefore, MNV-1 and TV, commonly used surrogates for human NoVs (Verhaelen et al., 2012; Hirneisen and Kniel, 2013a,b; Yang et al., 2018), were employed in this study. *Salmonella enterica* is a highly diverse bacterial species containing more than 2,600 different serovars differentiated by their antigenic presentation (Gal-Mor et al., 2014). This study selected a reference strain *Salmonella enterica* serovar Typhimurium strain SL 1344, which was used in multiple previous studies associated with fresh produce (Kroupitski et al., 2009; Delbeke et al., 2015b; Koukkidis et al., 2017) and two strains which were isolated from herbal plants (*Salmonella* *enterica* serovar Thompson strain RM1987 isolated from cilantro and *Salmonella enterica* serovar Thompson strain FMFP 899 isolated from basil).

The viruses are known to be able to survive for long periods on multiple fresh produce (Hirneisen and Kniel, 2013a; Wang et al., 2013). It was observed that NoVs cannot only attach firmly on the surface of fresh produce (Gandhi et al., 2010; Esseili et al., 2012a), but also be internalized into the plant tissues via different routes (Wei et al., 2011; DiCaprio et al., 2012). However, in this study, both MNV-1 and TV showed reductions to non-detectable levels after 3 days inoculated on the adaxial leaf surfaces of the 4 week old pre-harvest basil plants (> 5.5-log reductions for MNV-1 and > 3.3-log reductions for TV). This result indeed indicated higher virus infectivity reductions in comparison with previous reports. For instance, MNV-1 spiked on strawberries and raspberries lost only ca. 1-log infectivity after 3 days storage at 21°C (Verhaelen et al., 2012); MNV-1 inoculated on lettuce had about a 3.0-log drop in virus infectivity stored for 14 days at room temperature (Escudero et al., 2012); MNV-1 and TV inoculated on pre-harvest spinach adaxial leaf surfaces had decimal reduction times between 2 and 3 days (Hirneisen and Kniel, 2013a). Moreover, higher viral reductions were noticed on pre-harvest basil leaves than on fabric leaves after 3 days performed in parallel in this study as a control for both MNV-1 and TV. The reason can be due to the presence of antimicrobial/antiviral substances on the leaf surfaces of the actual pre-harvest basil plants (Suppakul et al., 2003; Hussain et al., 2008).

For *Salmonella enterica*, previously reports on the survival of *Salmonella* Newport (Gorbatsevich et al., 2013) and a mix of *Salmonella* Reading, Newport, and Typhimurium (Eckner et al., 2015) on growing (thus pre-harvest) basil plant leaves are available in literature. Despite of different experimental set-ups, both studies only observed a decline of *Salmonella* population on basil leaves and suggested a lack of growth. This is consistent with results of *Salmonella* Typhimurium SL 1344 and *Salmonella* Thompson RM1987 in the present study. However, for *Salmonella* Thompson FMFP 899, which was originally isolated from basil (Delbeke et al., 2015a), although over 3-log reductions were observed after 3 days, one out of three samples showed exceptional high levels after day 6 (5.5-log CFU/sample leaf) and day 9 (6.7-log CFU/sample leaf). Since this level still has not exceeded the initial inoculation level (7.3 ± 0.4-log CFU/sample leaf), the possibility that the bacteria simply survived well in certain shelters could not be ruled out. On the other hand, being different with human NoVs and their surrogates which are obligatory intracellular parasites, *Salmonella enterica* species have shown their capacity to cross a number of barriers requiring invasion of a large variety of cells and therefore could successfully infect hosts as diversified as animals or plants (Wiedemann et al., 2015). Numerous *Salmonella* genes have been identified as playing a role in its colonization of plant surfaces and tissues (Brandl et al., 2013). *Salmonella* cells from Arabidopsis leaf homogenates was shown to be as virulent as the inoculum grown in a nutrient-rich culture medium, invading the spleen and causing mortality in mice (Schikora et al., 2011). Previously, Brandl and Mandrell (2002) reported the fitness of *Salmonella* in the cilantro phyllosphere. They revealed that this pathogen has the ability to multiply and form microcolonies on leaves, although its population sizes are often exceeded by those of plant-associated bacterial species. Indeed, since the leaf surface is a harsh environment for bacteria due to UV radiation, the heterogeneity of nutrient availability and rapid fluctuations in temperature, and free water availability, *Salmonella* has been shown to preferentially move on leaves toward open stomata and colonize the vein areas, the bases of trichomes and damaged leaf areas, which may provide shelter and increase nutrient and water availability (Brandl et al., 2013; Wiedemann et al., 2015). Therefore, it is also quite possible that the *Salmonella* Thompson FMFP 899 in this study firstly decreased in population due to the environmental stress and afterward started to colonize and grew into high populations.

Internalization of enteric pathogens, both viruses and bacteria, into food crops has been increasingly recognized as one of the important mechanisms of produce contamination (Erickson, 2012; Hirneisen et al., 2012). Various levels of infectious viruses inoculated via roots were found in the edible parts of the leafy greens: up to 5 to 6-log PFU/g MNV-1 and TV in romaine lettuce (DiCaprio et al., 2012); 2.3 to 3.8-log PFU/g MNV-1 also in romaine lettuce (Wei et al., 2011); 4 to 5-log PFU/g MNV-1 in green onion and spinach (Hirneisen and Kniel, 2013b); ∼1-log PFU/g MNV-1 in kale and mustard microgreen (Wang and Kniel, 2016). As for *Salmonella*, 500 and 5,130 CFU/g of *Salmonella* Newport was detected in lettuce leaves with intact and damaged roots at 2 days post-inoculation of *Salmonella* in the soil but not 5 days later (Bernstein et al., 2007). Gorbatsevich et al. (2013) demonstrated that root internalization of *S. enterica* Newport into basil plants was plant-age dependent, while in all cases the internalized *Salmonella* survived only <30 h in the phyllosphere. In this study, indeed both infectious MNV-1 and *Salmonella* Thompson FMFP 899 were detected from the edible parts of basil after inoculation of the viruses and bacteria in plant growing medium, confirming the possibility of microbial internalization into food crops by root uptake. However, first of all, it must be noticed that very high microbial inoculation was used in the experimental set-up (8.46-log PFU/ml of MNV-1, 8.60-log CFU/ml of *S. enterica*), which is abnormal in the real-life on field contamination scenarios. Secondly, the infectivity of internalized MNV-1 and presence of *S. enterica* were only detected within 6 days after inoculation. The internalized *S. enterica* Thompson FMFP 899 was not quantifiable by direct enumeration, but was only positive after 24 h enrichment in buffered peptone water. In contrast, the *Salmonella* levels on the leaf surfaces were much more pronounced (up to 6.7-log CFU/sample leaf of *S. enterica* Thompson FMFP 899).

There could be concerns over the influence of virus recovery on the results of this study, as it is known that the minimal virus recovery efficiency requirement (1%) from fresh produce indicated in ISO/TS 15216 is in reality often not reached (Li et al., 2018). According to our preliminary tests, there was no significant loss of viruses (data not shown) probably because: (I) the sample sizes in this study are rather small (1 cm^2^ of leaf pieces in the survival test and 2 g of tissues in the internalization test, in comparison of 25 g of fresh produce in ISO/TS 15216); (II) the virus extraction procedure is simply grinding in buffer while the ISO/TS 15216 procedures include elution with agitation followed by precipitation with polyethylene glycol (PEG)/sodium chloride.

All in all, this study investigated the fate of two human NoV surrogates and three *S. enterica* strains on pre-harvest basil plants contaminated via both adaxial leaf surfaces and growth medium (thus roots). The results demonstrated the possible presence of high populations of *Salmonella* on basil leaves and therefore indicated the associated risks of causing human infection. In addition, this study supported our previous recommendation of drip or subsurface irrigation which could limit direct contact between edible plant tissue and irrigation water (splashes) and thus is less likely to introduce pathogens than furrow or sprinkler irrigation (Uyttendaele et al., 2015).

## Author Contributions

DL planned the experiments, performed the experiments, and wrote up the manuscript. MU oriented the research and did the final revision of the manuscript.

## Conflict of Interest Statement

The authors declare that the research was conducted in the absence of any commercial or financial relationships that could be construed as a potential conflict of interest.
